# Long-Term Survival and Death Causes of Systemic Lupus Erythematosus in China: A Systemic Review of Observational Studies

**DOI:** 10.1097/MD.0000000000000794

**Published:** 2015-05-01

**Authors:** Ziqian Wang, Yanhong Wang, Rongrong Zhu, Xinping Tian, Dong Xu, Qian Wang, Chanyuan Wu, Shangzhu Zhang, Jiuliang Zhao, Yan Zhao, Mengtao Li, Xiaofeng Zeng

**Affiliations:** From the Department of Rheumatology (ZW, RZ, XT, DX, QW, CW, SZ, JZ, YZ, ML, XZ), Peking Union Medical College Hospital, Peking Union Medical College & Chinese Academy of Medical Science, Key Laboratory of Rheumatology and Clinical Immunology, Ministry of Education, ; and Department of Epidemiology and Bio-statistics (YW), Institute of Basic Medical Sciences, China Academy of Medical Sciences & Peking Union Medical College, Beijing, China.

## Abstract

Systemic lupus erythematosus (SLE) is a chronic autoimmune disease with an increased risk of death compared to general population. Although previous studies showed improvement in survival of SLE, the long-term prognosis has not been elaborated in China.

This study aims to integrate the observational studies estimating current long-term survival of Chinese SLE patients and analyze the death-cause situation of SLE in China.

The study is a systemic review of English and non-English articles using MEDLINE, EMBASE, CNKI, WANFANG, and SINOMED databases. Additional studies were found by consultation with clinical experts, browse of references in selected papers, and search of related textbooks. Our major search terms were SLE, follow-up, prognosis, survival, mortality, and China.

We included cohort studies for survival analysis, and both cohort studies and case series for death-cause analysis in China.

The extraction of the articles were done by 2 authors independently using predesigned charts, including characteristics of study, clinical data, analyzing data, and study quality indicators.

All pooled analyses were conducted both for random-effects model and fixed-effects model. Funnel plots and Egger regression tests were applied to check potential publication bias. Heterogeneity was tested by sensitivity analysis. We identified 5 studies for survival analysis comprising 4469 Chinese patients with SLE (380 observed deaths). Thirty-six studies were suitable for death-cause analysis with 2179 observed deaths (derived from more than 20,000 Chinese patients with SLE). The overall pooled survival rates for SLE in China were 94% for 5-year survival rate and 89% for 10-year survival rate after disease onset from the year 1995 to 2013, which were similar with previous publications in Asia-Pacific area. The proportions of different causes of death showed infection (33.2%), renal involvement (18.7%), lupus encephalopathy (13.8%), and cardiovascular disease (11.5%) as the top 4 causes.

The overall survival rates for Chinese patients with SLE resembled previous publications in Asia-Pacific area. But the death causes of SLE in China were of some differences indicating relatively higher proportion of infection and lupus encephalopathy and lower cardiovascular disease. Ethnicity and more aggressive treatment might have contributed to the difference in death composition.

## INTRODUCTION

Systemic lupus erythematosus (SLE) is a kind of chronic multiorgan involved autoimmune disease which can be severe and fatal. With earlier diagnostic approaches and more appropriate treatment strategies, the survival of SLE has improved dramatically in last 50 years.^1^ The meta-analysis in 2012 demonstrated that the long-term survival had been improved between 1950s and 2000s, increasing from 74.8% to 94.8% for the 5-year survival and from 63.2% to 91.4% for the 10-year survival.^2^ However, the risk of death for SLE patients are still 2 times greater than those of general population (meta-standardized mortality ratio 2.98, 95% CI 2.32–3.83), which has driven further research on long-term survival and specific death causes of SLE patients.^3^

Previous epidemiological studies have found that the prevalence and incidence rates of Asian SLE patients were approximately 2 to 3 times higher than those of caucasians.^4–11^ Moreover, the Asian SLE patients were reported with higher clinical severity, significantly higher mean and maximum SLE disease activity index (SLEDAI), increased susceptibility to renal involvement, persistently active disease, and higher proportion of autoantibody positivity than non-Asian SLE patients.^12^ Many multiethnic cohort studies also showed that Asian patients are more likely to have severe SLE in many aspects, including increased renal disease, autoantibody positivity, disease activity, and organ damage accrual.^13–16^

China, as the largest country in Asia-Pacific area, was reported with relatively higher prevalence rate as 97.5 to 100/100,000 when compared to other ethnicities (generally 20–70/100,000).^9,17,18^ With increasing population in China, SLE is going to cause greater burden in the future. Unfortunately, there is limited information about the overall prognosis of Chinese SLE patients now. Therefore, the aim of this study is to focus on the long-term prognosis of SLE patients to review the pictures of their 5- and 10-year long-term survivals as well as major causes of death in China.

## METHODS

### Eligibility and Exclusion Criteria

The criteria of eligibility for long-term survival was defined as retrospective or prospective cohort studies of SLE patients who were diagnosed by a recognized criteria such as the criteria of American College of Rheumatology (ACR) in 1982 or 1997, with report of the long-term survival rates (5- or 10-year survival rate) measured by Kaplan-Meier analysis. The criteria for major death cause of SLE was defined to include all the studies with death-cause information in which those SLE patients were diagnosed by a recognized criteria such as the criteria of ACR in 1982 or 1997, without limitation in the types of studies. In addition, the studies that only included a special subgroup of SLE patients (such as early onset, late onset, lupus nephritis, etc.) were excluded due to their low generality.

### Literature Search

We searched the literatures published in MEDLINE (OVID 1946–January 2015), EMBASE (1980–January 2015), WANFANG (1998–January 2015), CNKI (1994–January 2015), and SINOMED (1978–January 2015). No special search software was used. We also hand searched the references of selected literatures, related textbooks, and consulted with professionals. For the unpublished research and papers that we could not find full texts, we also tried to contact with the authors. The search terms were as follows: SLE, prognosis, follow-up, survival, mortality, China, Chinese, Asia, Taiwan, Hong Kong, and Han. No language or publication status restrictions were imposed. We took the following searching flowchart used in MEDLINE (OVID) as an example:

1. (systemic lupus erythematosus or lupus erythematosus or lupus).ti,ab.;
2. (prognosis or outcome or follow up or long term or fatality or mortality or death or morbidity or survival rate).ti,ab.;
3. 1 and 2;
4. (China or Chinese or Taiwan or han or Hong Kong or Asia or Asian or orient^∗^).ti,ab,sh.;
5. 3 and 4.

### Study Selection

Study inclusion and eligibility assessment were conducted by 2 reviewers independently. Disagreements during the process were discussed by the 2 reviewers and then determined with a third reviewer.

### Data Collection Process

We designed a data extracting chart according to the Cochrane Handbook as well as the demands in this study. In order to refine the extracting chart, the pilot test was done by 5 articles randomly chosen from all the included studies. Two reviewers independently extracted information from the included studies. The differences were discussed by them together and ultimately determined with a third reviewer.

### Data Items

Information were extracted from all included studies on characteristics of the patients, including total patient number, gender proportion, average age of disease onset, average age of death, and course of disease; type of treatment if available; 5- and 10-year survival rates by Kaplan–Meier analysis; and major death causes and counts.

### Assessment on the Quality of Studies

For meta-analysis of long-term survival, to assess the quality of studies, a 10-score scale was designed based on the scale used in previous publications, which was also used in formerly published meta-analysis of observational studies on SLE.^3,19,20^ This scale contains 5 items counting with 0, 1, or 2 points: sample type (no definition = 0, clinic-based sample = 1, community-based sample = 2); information of defaulters (not mention = 0, only rates of lost to follow-up = 1, elucidation of both rates and reasons of lost to follow-up = 2); diagnosis of SLE (nonvalidated criteria = 0, other validated criteria = 1, the criteria of ACR for SLE = 2); methods to confirm death (not given = 0, predefined but nonvalidated criteria = 1, predefined and validated criteria = 2); time of exposure to SLE (<5 years = 0, 5–10 years = 1, >10 years = 2). Two independent reviewers completed the quality assessment, respectively, and a third reviewer helped to determine when disagreement appeared. The studies that were ≥7 points were classified as high-quality studies, and the rest were regarded as studies with low quality.

### Statistical and Bias Analysis

Survival rates were extracted directly from Kaplan–Meier analysis or curves. The logit model was used to transform the extracted rates. Then, the pooled logits were calculated and transformed back into survival rates for better interpretations and shown in forest plots. Sensitivity analysis by omitting each study was conducted if there was heterogeneity across the studies. The potential publication bias was examined via funnel plots and Egger regression tests. The R project was employed for statistical computing.

In order to picture the cause of death for SLE patients in China, the studies were classified into 3 groups in order to show the general situation in different time. Group I included all the studies with SLE patients observed by the year 2000. Group II included studies that involved SLE patients since 2000 or after, and others were in group III. The deaths were accumulated, and the percentages of different causes of death in total, group I and II, were calculated. All numerical analysis and graphics were performed by SPSS 19.0.

## RESULTS

### Search of Study

**FIGURE 1:**
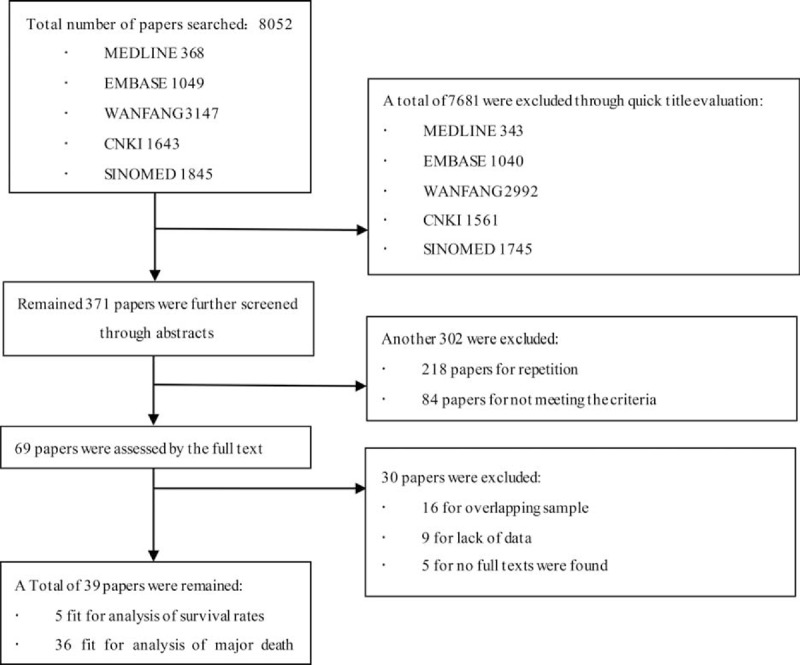
The flowchart for the result of literature search.

A total of 8052 papers were initially spotted out, 7681of them were excluded by title screening for no relation with survival or death causes. During abstract viewing, another 302 studies were excluded. Finally, 69 papers were remained for full-paper evaluation, and only 39 were fit for our selection criteria and contained all the information we needed to extract. Among them, 5 studies contained enough survival information for meta-analysis. There were 36 studies in total that were suitable for cause of death analysis. The process was shown as flow chart in Figure 1.

### Study Characteristics

There were 5 studies included in meta-analysis of long-term survival with high quality, involving a total of 4469 SLE patients.^21–25^ The characteristics of these studies were shown in Table 1. In addition, 36 studies were involved in death-cause analysis, of which 2 studies were cohort studies used for meta-analysis of survival, and others were case series from different hospitals. A total of 2179 death cases from 1950 to 2013 were reported in these included studies.

**TABLE 1:**
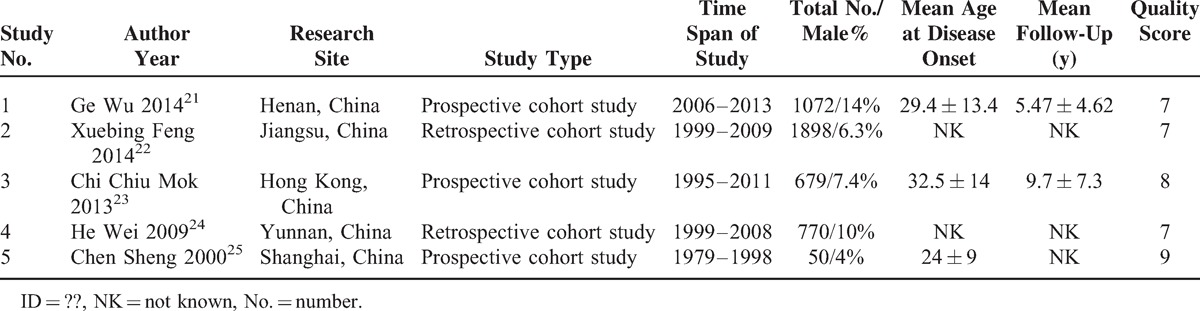
Characteristics of Included Studies for Survival Rate Analysis

### The Overall 5 and 10-Year Survival Rates

**FIGURE 2:**
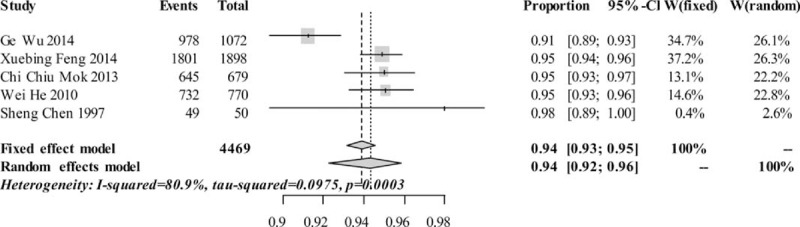
Pooled 5-year survival rates.

**FIGURE 3:**
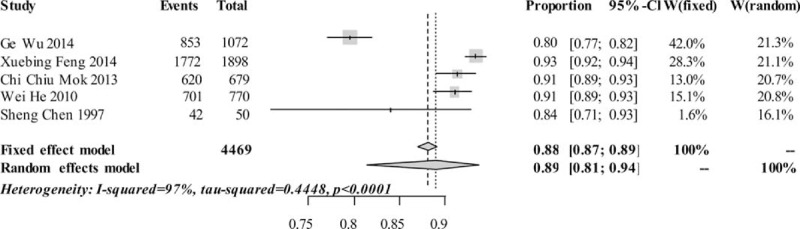
Pooled 10-year survival rates.

The pooled 5-year survival rates were 0.9389 [0.9314, 0.9457] and 0.9431 [0.9225, 0.9585] on fixed-effects model and random-effects model, respectively (see Figure 2). The pooled 10-year survival rates were 0.8810 [0.8705, 0.8907] and 0.8893 [0.8141, 0.9365] on fixed-effects model and random-effects model, respectively (see Figure 3). The estimated heterogeneity were [I^2^ 80.9%, τ^2^ 0.0975, *P* = 0.0003] and [I^2^ 97%, τ^2^ 0.4448, *P* < 0.0001], respectively, which means that the survival rates differed among those studies.

We conducted sensitivity analysis by omitting each study to find the potential source of heterogeneity. The study by Wu et al (2014) accounted for more heterogeneity than other studies, but it did not make a great difference in survival rates when omitted it.^21^ After retrospective reviewing on these studies, we found the study by Wu et al had a relatively shorter period of follow-up. Moreover, even though this study was a prospective study that involved a larger group of patients, it presented a relatively higher proportion of defaulters (about 37%). All of these might explain the reason why the survival rates in the study by Wu et al were relatively lower than the other studies. Limited by the study number, we were not able to conduct metaregression analysis to further assess the source of bias.

### Publication Bias Analysis

The funnel plots for the 5- and 10-year survival rates were both generally symmetric. Results of Egger regression test were [t = 0.801, df = 3, bias = 1.94, *P* value = 0.4817] for 5-year survival rates, and [t = 0.4711, df = 3, bias = 3.46, *P* value = 0.6697] for 10-year survival rates, implying that publication bias was small or might not be the major contributor for heterogeneity across the studies.

### Death-Cause Analysis

**FIGURE 4:**
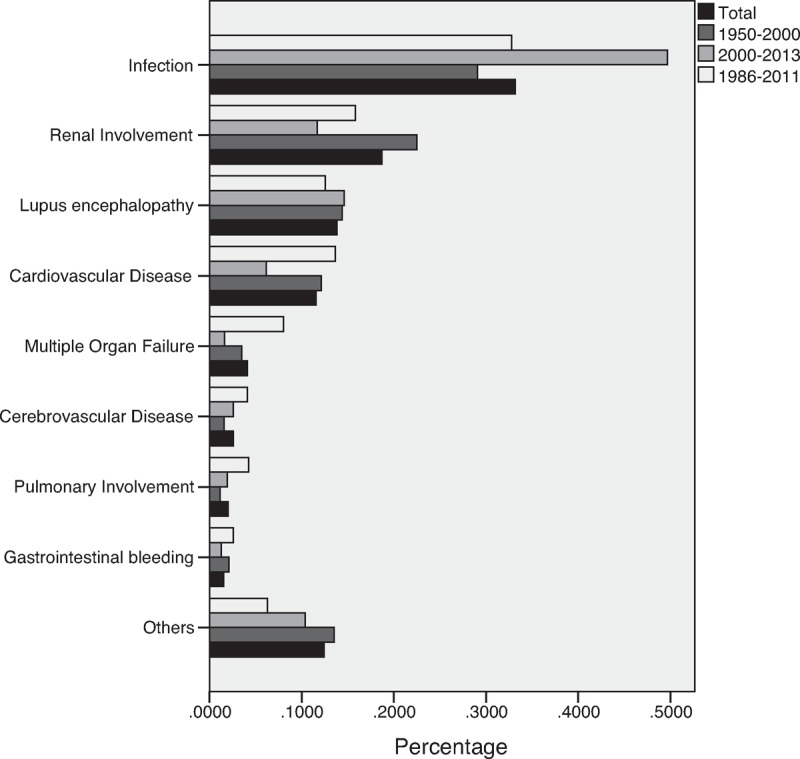
The proportion of different death causes.

A total of 36 studies were involved for further analysis of major death causes of SLE (see Table 2). Among them, 19 studies were classified in group I,^25–43^ 6 studies were in group II,^42,44–48^ and 12 studies were included in group III.^23,49–59^ Among them, the study by Fei et al was both in group I and group II because it provided information from different times.^42^ A total of 2179 deaths were involved, 929 deaths in the group of 1950 to 1999 and 308 in the 2000 to 2013 group. The top 8 major death causes for the 2 groups were almost the same, except for some changes in the proportions. The percentages of different causes of death in total deaths and 3 groups were infection [33.2%, 29.1%, 49.7%, 32.7%], renal involvement [18.7%, 22.5%, 11.7%, 15.8%], lupus encephalopathy [13.8%, 14.4%, 14.6%, 12.6%], cardiovascular disease (CVD) [11.5%, 12.1%, 6.17%, 13.6%], multiple organ failure [4.13%, 3.51%, 1.62%, 8.05%], cerebrovascular disease [2.57%, 1.58%, 2.60%, 4.09%], pulmonary involvement [2.02%, 1.14%, 1.95%, 4.23%], and gastrointestinal bleeding [1.56%, 1.35%, 1.30%, 2.59%] (see Figure 4). Apart from all these causes, each of other causes led to less than 1% of the deaths, such as liver involvement (0.5%) and cancer (0.7%), etc.

**TABLE 2:**
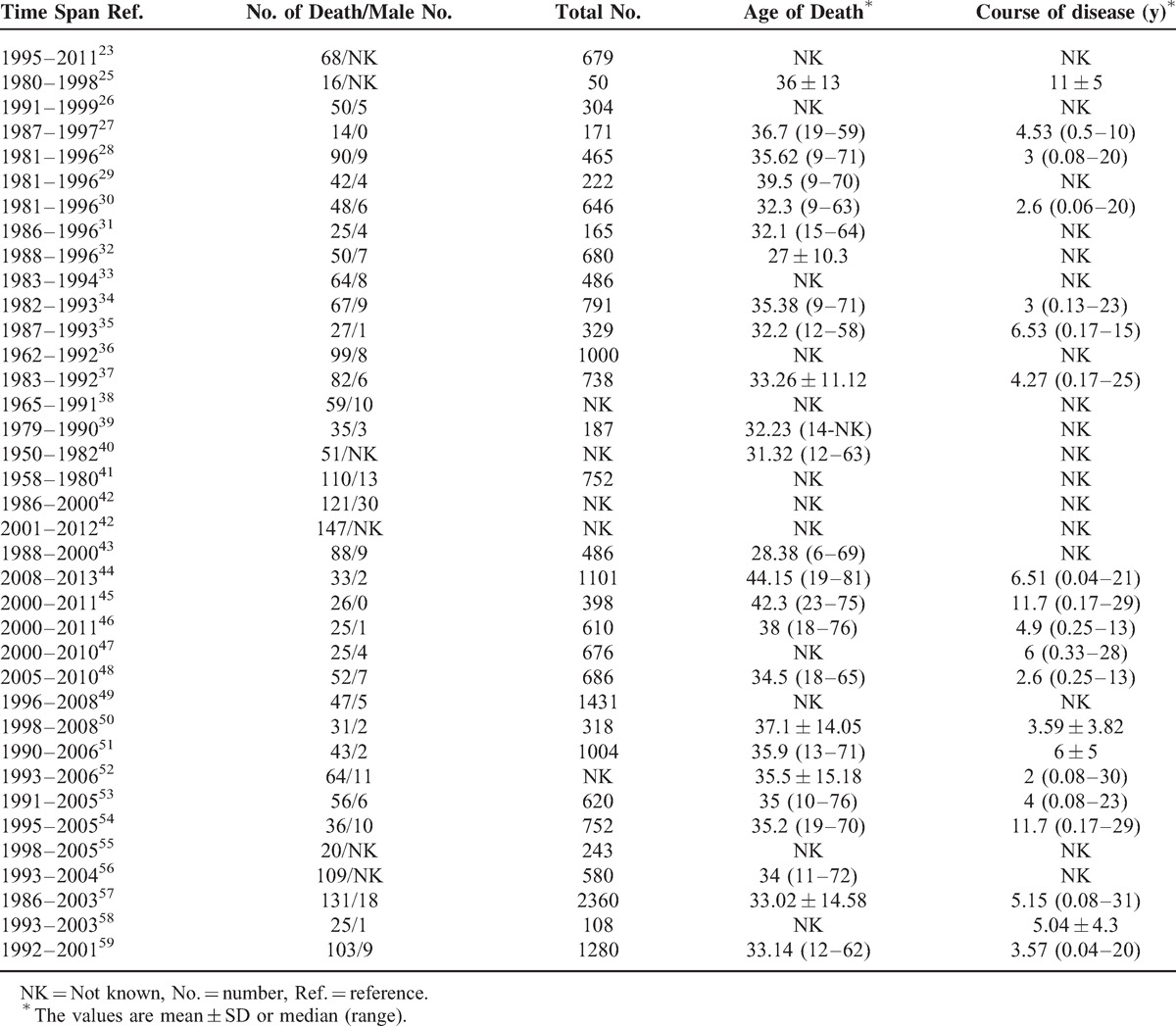
Basic Information of Studies Involved for Death-Cause Analysis

## DISCUSSION

Although with the limitation of the number of included studies and heterogeneity existed across the studies, this study gave us rough pooled long-term survival rates for SLE in China, 94% for 5-year survival rate and 89% for 10-year survival rate after disease onset from 1995 to 2013. A prospective study from 1995 to 2010 in a Danish community reported similar cumulative survival rates as 93.6% and 86.5% for 5- and 10-year survival rates, respectively.^60^ In 2012, Mak et al published a meta-analysis for global trend of SLE from 1990s to 2000s and reported 92.67% to 94.79% for pooled 5-year survival rate and 88.08% to 91.41% for 10-year survival rate, which were similar with our results.^2^ The study by Mak et al also found that the pooled survival rates of Asian studies (88% for 5-year survival rate and 84% for 10-year survival rate) were relatively lower than those of Europe (93% for 5-year survival rate and 91% for 10-year survival rate). Apart from less advanced medical technology, more severe clinical manifestations and poorer prognosis might have contributed to the gap of survival between Asians and Caucasians.^15,61^ However, most of the Asian studies included in the meta-analysis by Mak et al were before 1990s and some of recent studies were not covered, so that the pooled survival rates were poorer than those in our study.^2^

In 2014, Thomas et al reported that the top leading death causes of SLE in France between 2000 and 2009 were CVD, neoplasm, infection, cerebrovascular disease, respiratory disease, and renal disease.^62^ The cohort study in Canadian also found the similar order of major death causes.^63^ However, our results showed a higher proportion of infection, fewer of CVD, and higher percentage of lupus encephalopathy, which were different from those in former studies.

Remarkably, we found that infection was the top 1 cause of death among the SLE patients in China. The infection of Chinese SLE patients was positively correlated with the use of prednisone and immunosuppressive agents, so more aggressive treatment for SLE may play an important role, which might be associated with severer disease and higher disease activity for SLE in China.^64,65^

CVD was no more than 20% of the death causes in our results, even combined with cerebrovascular disease. Latest reports on the burden of CVD have not found ethnic differences among the general population in different countries,^66–68^ so the relatively low level of CVD could be associated with SLE-related conditions. Lower dose steroids combined with less aggressive immunosuppressive agents were reported to increase the risk of carotid plaques in SLE patients, so aggressive control of the disease may help reduce the risk of CVD.^69,70^ Thus the aggressive treatment in China, high dose of steroids and prevalent use of immunosuppressive agents, might be the key factor for the relatively fewer deaths of CVD, and on the other hand, contributed to the increase of infection. In addition, prospective cohort studies have shown that hydroxychloroquine can help reduce LDL-C levels and protect from CVD.^71,72^ In this case, additional use of antimalarial in maintenance may assist in not only reducing infection but also preventing from CVD in the future.

Our results implied that there might be more deaths of lupus encephalopathy in Chinese SLE patients. The percentage of neuropsychiatric lupus in a Chinese cohort was about 4.8% to 12.2%, a little lower compared to about 20% to 40% in other SLE cohorts.^73–76^ However, the proportions of acute confusional state (18.7%) and seizures (30.5%) in this paper were much higher than other published studies (3.7% and 8.3%, respectively).^75,77,78^ Another 2 cohorts in Asia (Hong Kong and Thailand) also reported relatively higher proportions of acute confusional state (14% and 11.2%) and seizures (28% and 54.1%).^79,80^ Although these results were related to more severe course of inpatients, it did indicate that they partly contribute to the higher proportion of death for lupus encephalopathy in China, and even in Asia.

In our study, pulmonary involvement is one of the major death causes for SLE. Pulmonary involvement is more common for SLE compared with other connective tissue diseases, whereas is with mild manifestations in most situations.^81^ However, several critical complications, rare but with high mortality rates and predictive for poor prognosis, might cause death and are often ignored by clinician, including pulmonary arterial hypertension (PAH), diffuse alveolar hemorrhage (DAH), and interstitial lung disease (ILD).^82^ The Chinese SLE Treatment and Research group (CSTAR) registry, the first multicenter registry in China, has reported prevalence rates of PAH and ILD as 3.8% and 4.2%.^76,83^ Unlike western patients, the most common reason for connective tissue disease (CTD)–associated PAH in Chinese is SLE (49%), and the mortality rate is markedly increased compared to general SLE patients.^84^ ILD, including acute lupus pneumonitis and chronic interstitial pneumonitis, contributes to poor long-term prognosis for SLE patients.^85^ For DAH, which is even rarer (prevalence rate about 1.4%–1.9%), was reported to have mortality rate as high as 62% to 66.7% in China,^82,86^ similar with that in a Korean study (61.9%).^87^ After all, we should pay more attention to these diseases for Chinese SLE patients.

The heterogeneity across the studies in survival rate analysis was the major limitation of our study. Besides the number of studies and the study by Wu et al, baseline characteristics of patients and wide spectrum of treatment strategies should be the major contributors for heterogeneity. In addition, more case series were included in death cause analysis of SLE, so we just accumulated the results of different studies, neglecting the possible difference of different clinic cohorts and the background treatment strategies.

## CONCLUSIONS

The condition of SLE is improving now in China. Our results showed a comparable general long-term prognosis to other ethnicities. However, the proportions of death causes were a little different with other countries, which might be related to ethnic differences and the more aggressive treatment strategy in China. After all, the next step for the rheumatologists in China should be on seeking for the reason to cause ethnicity for SLE in China, the balance between treatment and potential impacts, and alternative therapies to further improve long-term survival.

## References

[1] Doria A Gatto M Zen M Optimizing outcome in SLE: treating-to-target and definition of treatment goals. *Autoimmun Rev* 2014; 13:770–777. 24480071 10.1016/j.autrev.2014.01.055

[2] Mak A Cheung MWL Chiew HJ Global trend of survival and damage of systemic lupus erythematosus: meta-analysis and meta-regression of observational studies from the 1950s to 2000s. *Semin Arthritis Rheum* 2012; 41:830–839. 22257558 10.1016/j.semarthrit.2011.11.002

[3] Yurkovich M Vostretsova K Chen W Overall and cause-specific mortality in patients with systemic lupus erythematosus: a meta-analysis of observational studies. *Arthritis Care Res* 2014; 66:608–616. 10.1002/acr.22173 24106157

[4] Ferucci ED Johnston JM Gaddy JR Prevalence and incidence of systemic lupus erythematosus in a population-based registry of American Indian and Alaska Native people, 2007-2009. *Arthritis Rheumatol* 2014; 66:2494–2502. 24891315 10.1002/art.38720 PMC4617772

[5] Lim SS Bayakly AR Helmick CG The Incidence and Prevalence of Systemic Lupus Erythematosus, 2002-2004: the Georgia Lupus Registry. *Arthritis Rheumatol* 2014; 66:357–368. 24504808 10.1002/art.38239 PMC4617771

[6] See L Kuo C Chou I Sex- and age-specific incidence of autoimmune rheumatic diseases in the Chinese population: a Taiwan population-based study. *Semin Arthritis Rheum* 2013; 43:381–386. 23916348 10.1016/j.semarthrit.2013.06.001

[7] Jakes RW Bae S Louthrenoo W Systematic review of the epidemiology of systemic lupus erythematosus in the Asia-Pacific region: prevalence, incidence, clinical features, and mortality. *Arthritis Care Res* 2012; 64:159–168. 10.1002/acr.20683 22052624

[8] Flower C Hennis AJM Hambleton IR Systemic lupus erythematosus in an Afro-Caribbean population: incidence, clinical manifestations and survival in the Barbados national lupus registry. *Arthritis Care Res* 2012; 64:1151–1158. 10.1002/acr.21656 22392730

[9] Pons-Estel GJ Alarcón GS Scofield L Understanding the Epidemiology and Progression of Systemic Lupus Erythematosus. *Semin Arthritis Rheum* 2010; 39:257–268. 19136143 10.1016/j.semarthrit.2008.10.007 PMC2813992

[10] Mok CC To CH Hod LY Incidence and mortality of systemic lupus erythematosus in a southern Chinese population, 2000-2006. *J Rheumatol* 2008; 35:1978–1982. 18688913

[11] Mok C Epidemiology and survival of systemic lupus erythematosus in Hong Kong Chinese. *Lupus* 2011; 20:767–771. 21148605 10.1177/0961203310388447

[12] Golder V Connelly K Staples M Association of Asian ethnicity with disease activity in SLE: an observational study from the Monash Lupus Clinic. *Lupus* 2013; 22:1425–1430. 23942610 10.1177/0961203313500547

[13] Jakes RW Bae S Louthrenoo W Systematic review of the epidemiology of systemic lupus erythematosus in the Asia-Pacific region: prevalence, incidence, clinical features, and mortality. *Arthritis Care Res* 2012; 64:159–168. 10.1002/acr.20683 22052624

[14] Connelly K Morand EF Hoi AY Asian ethnicity in systemic lupus erythematosus: an Australian perspective. *Intern Med J* 2013; 43:618–624. 23279565 10.1111/imj.12070

[15] Ong C Nicholls K Becker G Ethnicity and lupus nephritis: an Australian single centre study. *Intern Med J* 2011; 41:270–278. 21426464 10.1111/j.1445-5994.2009.02159.x

[16] Cervera R Khamashta MA Font J Systemic lupus erythematosus: clinical and immunologic patterns of disease expression in a cohort of 1000 patients. The European Working Party on Systemic Lupus Erythematosus. *Medicine (Baltimore)* 1993; 72:113–124. 8479324

[17] Mok CC Epidemiology and survival of systemic lupus erythematosus in Hong Kong Chinese. *Lupus* 2011; 20:767–771. 21148605 10.1177/0961203310388447

[18] Yeh KW Yu CH Chan PC Burden of systemic lupus erythematosus in Taiwan: a population-based survey. *Rheumatol Int* 2013; 33:1805–1811. 23314932 10.1007/s00296-012-2643-6

[19] Takkouche B Etminan M Montes-Martinez A Personal use of hair dyes and risk of cancer: a meta-analysis. *JAMA* 2005; 293:2516–2525. 15914752 10.1001/jama.293.20.2516

[20] Bhutta AT Cleves MA Casey PH Cognitive and behavioral outcomes of school-aged children who were born preterm: a meta-analysis. *JAMA* 2002; 288:728–737. 12169077 10.1001/jama.288.6.728

[21] Wu G Jia X Gao D Survival rates and risk factors for mortality in systemic lupus erythematosus patients in a Chinese center. *Clinical Rheumatol* 2014; 33:947–953. 10.1007/s10067-014-2596-0 24794488

[22] Feng X Zou Y Pan W Associations of clinical features and prognosis with age at disease onset in patients with systemic lupus erythematosus. *Lupus* 2014; 23:327–334. 24297642 10.1177/0961203313513508

[23] Mok CC Chan PT Ho LY Prevalence of the antiphospholipid syndrome and its effect on survival in 679 Chinese patients with systemic lupus erythematosus: a cohort study. *Medicine (United States)* 2013; 92:217–222. 10.1097/MD.0b013e31829cae47 PMC4553973 23793109

[24] Wei H Hong X Mei C Survival rates and associated risk factors for systemic lupus erythematosus in Yunnan province (Chinese). Paper presented at: the 15th National Conference on Dermatovenereology of Chinese Medical Association; 2009; Tianjin, China.

[25] Sheng C Shunle C An eighteen-year follow-up study of systemic lupus erythematosus (Chinese). *Chinese J Rheumatol* 2000; 4:27–31.

[26] Yingying Z Jiansheng W Zhonghe L Analysis on cause death of 50 patients with systemic lupus erythematosus (Chinese). *Central Plains Med J* 2000; 27:18–19.

[27] Yong C Death cause analysis of systemic lupus erythematosus (Chinese). *Ningbo Medical* 1998; 10:172.

[28] Ciguang M Daoyou Z Rengao Y Analysis of causes of death in 90 patients with systemic lupus erythematosus (Chinese). *Chinese J Nephrol* 1998; 14:114–116.

[29] Ruijun L Kuo W Zhong L Analysis of the death cause on 42 dead cases with systemic lupus erythemalosus (Chinese). *J Dali Med Coll* 2000; 9:11–12.

[30] Zhongyi C Analysis of death causes on 48 dead cases with systemic lupus erythematosus (Chinese). *Fujian Med J* 2006; 28:55–57.

[31] Linjie C Zhijun L Weidong C Cause of death analysis of 25 cases with systemic lupus erythematosus (Chinese). *J Bengbu Med Coll* 1997; 22:97.

[32] Danhong Y Rengao Y Death cause analysis of 50 systemic lupus erythematosus patients (Chinese). *J Postgrad Med* 1998; 21:249–250.

[33] Shangbei S Analysis of causes in 64 death cases with systemic lupus erythematosus (Chinese). *Chin J Derm Venereol* 1997; 11:27–28.

[34] Zhiling C Xingqi Z Analysis of the causes of death in 67 systemic lupus erythematosus patients (Chinese). *J Clin Dermatol* 1996; (02).

[35] Shufan L Lijuan Z Death cause analysis on patients with systemic lupus erythematosus (Chinese). *Liaoning Med J* 1996; (03).

[36] Xueyi J Xiazhe F Xueli Y Clinical study of 1000 cases with systemic lupus erythematosus (Chinese). *Chinese J Dermatovenerol* 1993; (02):76-79.

[37] Huanqin C Tiezheng T Fengshan Z Analysis of death cases with systemic lupus erythematosus (Chinese). *J Harbin Med Univ* 1995; (03).

[38] Zhongying W Feng L Ping Z Analysis of common causes in patients with systemic lupus erythematosus (Chinese). *Jilin Med J* 1991; (05).

[39] Xiangpei L Xiaomei L Cause of death analysis on 35 systemic lupus erythematosus patients (Chinese). *Anhui Med J* 1994; (02).

[40] Qin W Qian W Xiaofeng Z Analysis of death causes in 187 patients with systemic lupus erythematosus (Chinese). *China Med* 2008; 3:598–599.

[41] Shouyi S Death cause analysis of 110 cases in systemic lupus erythematosus (Chinese). *Chinese J Dermatol* 1984; 3:153–156.

[42] Fei Y Shi X Gan F Death causes and pathogens analysis of systemic lupus erythematosus during the past 26 years. *Clinical Rheumatol* 2014; 33:57–63. 10.1007/s10067-013-2383-3 24046218

[43] Liya Y Bang A Huijie L Death cause analysis of 88 systemic lupus erythematosus patients (Chinese). *Henan Med Info* 2002; 10:20–21.

[44] Tao S Weiguo X Cause of death analysis on 33 cases of systemic lupus erythematosus (Chinese). *J China Med Univ* 2014; 43:850–851.

[45] Weiwei C Xiao S Analysis of the death causes in 26 systemic lupus erythematosus patients (Chinese). *J Clin Intern Med* 2012; 29:494.

[46] Jin-xiong H Min W Le W The cause of death analysis in patient with systemic lupus erythematosus (an analysis of 25 cases) (Chinese). *Clin Misdiag Misther* 2012; 25:92–94.

[47] Fengjiao G Wencheng Q Analysis on the death-associated factors in 25 systemic lupus erythematosus patients (Chinese). *New Med* 2012; 43:38–41.

[48] Jinmei Z Jing Y Yi L Infection of patients died from systemic lupus erythematosus (Chinese). *Med J West China* 2011; 23:356–358.360.

[49] Jiafeng S Xiangpei L Jinhui T Analysis of death causes in 47 patients with systemic lupus erythematosus (Chinese). *Anhui Med J* 2010; 31:768–770.

[50] Xuejun H Qinglian O Xueqiong L Analysis of the causes of death and associated factor of 31 systemic lupus erythematosus patients (Chinese). *China Clin Prac Med* 2010; 04:63–64.

[51] Danqi D Hong X Haiqiong Y Cause of death analysis in 43 systemic lupus erythematosus patients (Chinese). *Chinese J Rheumatol* 2008; 12:285.

[52] Lin P *Causes and Risk Factors Associated with Death of 64 Patients With Systemic Lupus Erythematosus: A Retrospective Case-Control Study* (Chinese) [master's thesis]. Department of Dermatology and Venereology, Fudan University; 2007.

[53] Guan-ling Z Qiu-sheng Z Clinic analysis of death causes in 56 patients with systemic lupus erythematosus (Chinese). *Appl J Gen Pract* 2007; 5:604–605.

[54] Ling L Xiangui C Analysis of death causes in 36 patients with systemic lupus erythematosus (Chinese). Paper presented at: the Fourth Annual Conference of Dermatovenereology in Western China; 2006, Urumchi, China.

[55] Li-min R Hua YE Jin-xia Z Analysis of 5-year survival rate and prognostic indicators of systemic lupus erythematosus (Chinese). *Chinese J Rheumatol* 2009; 13:156–158.

[56] Weifang Z *Analysis of the Survival Rate and Related Factors in Hospitalized Systemic Lupus Erythematosus Patients* (Chinese) [master's thesis]. Department of Dermatology and Venereology, Zhejiang University; 2006.

[57] Yijin L Fanqin Z Guozhen T Analysis on the causes of death in 131 systemic lupus erythematosus patients (Chinese). *Guangdong Med* 2004; 25:689–690.

[58] Wen-Hui H Yi T Na YU A 10-year analysis of the causes of death and associated factors in systemic lupus erythematosus (Chinese). *Acad J Guangzhou Med Coll* 2004; 32:42–44.

[59] Zhanquan H Xiaodong W Ji C Death cause analysis in patients with systemic lupus erythematosus (Chinese). *Bull Med Res* 2003; 32:41–42.

[60] Voss A Laustrup H Hjelmborg J Survival in systemic lupus erythematosus, 1995–2010. A prospective study in a Danish community. *Lupus* 2013; 22:1185–1191. 23873432 10.1177/0961203313498796

[61] Kuan WP Li EK Tam LS Lupus organ damage: what is damaged in Asian patients? *Lupus* 2010; 19:1436–1441. 20947554 10.1177/0961203310370050

[62] Thomas G Mancini J Jourde-Chiche N Mortality associated with systemic lupus erythematosus in France assessed by multiple-cause-of-death analysis. *Arthritis Rheumatol* 2014; 66:2503–2511. 24910304 10.1002/art.38731

[63] Bernatsky S Boivin JF Joseph L Mortality in systemic lupus erythematosus. *Arthritis Rheum* 2006; 54:2550–2557. 16868977 10.1002/art.21955

[64] Xu G Liu M Yu K A prospective study of nosocomial infection in patients with systemic lupus erythematosus. *Chin J Rheumatol* 2003; 7:216–219.

[65] Danza A Ruiz-Irastorza G Infection risk in systemic lupus erythematosus patients: susceptibility factors and preventive strategies. *Lupus* 2013; 22:1286–1294. 24098001 10.1177/0961203313493032

[66] Nichols M Townsend N Scarborough P Cardiovascular disease in Europe 2014: epidemiological update. *Eur Heart J* 2014; 35:2950–2959. 25139896 10.1093/eurheartj/ehu299

[67] National Center for Cardiovascular Disease. Report on Cardiovascular Diseases in China (2013). China: Encyclopedia of China Publishing House; 2013.

[68] de Fatima Marinho De Souza M Gawryszewski VP Ordunez P Cardiovascular disease mortality in the Americas: current trends and disparities. *Heart* 2012; 98:1207–1212. 22826558 10.1136/heartjnl-2012-301828

[69] Gustafsson JT Svenungsson E Definitions of and contributions to cardiovascular disease in systemic lupus erythematosus. *Autoimmunity* 2014; 47:67–76. 24228980 10.3109/08916934.2013.856005

[70] Magder LS Petri M Incidence of and risk factors for adverse cardiovascular events among patients with systemic lupus erythematosus. *Am J Epidemiol* 2012; 176:708–719. 23024137 10.1093/aje/kws130 PMC3571250

[71] Cairoli E Rebella M Danese N Hydroxychloroquine reduces low-density lipoprotein cholesterol levels in systemic lupus erythematosus: a longitudinal evaluation of the lipid-lowering effect. *Lupus* 2012; 21:1178–1182. 22641182 10.1177/0961203312450084

[72] Jung H Bobba R Su J The protective effect of antimalarial drugs on thrombovascular events in systemic lupus erythematosus. *Arthritis Rheum* 2010; 62:863–868. 20131232 10.1002/art.27289

[73] Borowoy AM Pope JE Silverman E Neuropsychiatric lupus: the prevalence and autoantibody associations depend on the definition: results from the 1000 faces of lupus cohort. *Semin Arthritis Rheum* 2012; 42:179–185. 22595642 10.1016/j.semarthrit.2012.03.011

[74] Hanly JG Urowitz MB Su L Prospective analysis of neuropsychiatric events in an international disease inception cohort of patients with systemic lupus erythematosus. *Ann Rheum Dis* 2010; 69:529–535. 19359262 10.1136/ard.2008.106351 PMC2929162

[75] Zhou H Zhang F Tian X Clinical features and outcome of neuropsychiatric lupus in Chinese: analysis of 240 hospitalized patients. *Lupus* 2008; 17:93–99. 18250131 10.1177/0961203307085671

[76] Li M Zhang W Leng X Chinese SLE treatment and Research group (CSTAR) registry: I. Major clinical characteristics of Chinese patients with systemic lupus erythematosus. *Lupus* 2013; 22:1192–1199. 23963101 10.1177/0961203313499086

[77] Hanly JG McCurdy G Fougere L Neuropsychiatric events in systemic lupus erythematosus: attribution and clinical significance. *J Rheumatol* 2004; 31:2156–2162. 15517627

[78] Sanna G Bertolaccini ML Cuadrado MJ Neuropsychiatric manifestations in systemic lupus erythematosus: prevalence and association with antiphospholipid antibodies. *J Rheumatol* 2003; 30:985–992. 12734893

[79] Mok CC Lau CS Wong RW Neuropsychiatric manifestations and their clinical associations in southern Chinese patients with systemic lupus erythematosus. *J Rheumatol* 2001; 28:766–771. 11327248

[80] Kasitanon N Louthrenoo W Piyasirisilp S Neuropsychiatric manifestations in Thai patients with systemic lupus erythematosus. *Asian Pac J Allergy Immunol* 2002; 20:179–185. 12587842

[81] Keane MP Lynch JR Pleuropulmonary manifestations of systemic lupus erythematosus. *Thorax* 2000; 55:159–166. 10639536 10.1136/thorax.55.2.159 PMC1745678

[82] Shen M Wang Y Xu WB Pleuropulmonary manifestations of systemic lupus erythematosus (Chinese). *Chinese Medical Journal* 2005; 85:3392–3395. 16409858

[83] Li M Wang Q Zhao J Chinese SLE Treatment and Research group (CSTAR) registry: II. Prevalence and risk factors of pulmonary arterial hypertension in Chinese patients with systemic lupus erythematosus. *Lupus* 2014; 23:1085–1091. 24651670 10.1177/0961203314527366

[84] Hao YJ Jiang X Zhou W Connective tissue disease-associated pulmonary arterial hypertension in Chinese patients. *Eur Respir J* 2014; 44:963–972. 24791829 10.1183/09031936.00182813

[85] Cheema GS Quismorio FJ Interstitial lung disease in systemic lupus erythematosus. *Curr Opin Pulm Med* 2000; 6:424–429. 10958234 10.1097/00063198-200009000-00007

[86] Shen M Zeng X Tian X Diffuse alveolar hemorrhage in systemic lupus erythematosus: a retrospective study in China. *Lupus* 2010; 19:1326–1330. 20647253 10.1177/0961203310373106

[87] Kwok SK Moon SJ Ju J Diffuse alveolar hemorrhage in systemic lupus erythematosus: risk factors and clinical outcome: results from affiliated hospitals of Catholic University of Korea. *Lupus* 2011; 20:102–107. 20956464 10.1177/0961203310381511

